# Religious Fundamentalism Modulates Neural Responses to Error-Related Words: The Role of Motivation Toward Closure

**DOI:** 10.3389/fpsyg.2018.00285

**Published:** 2018-03-27

**Authors:** Małgorzata Kossowska, Paulina Szwed, Miroslaw Wyczesany, Gabriela Czarnek, Eligiusz Wronka

**Affiliations:** Department of Philosophy, Institute of Psychology, Jagiellonian University, Kraków, Poland

**Keywords:** religious fundamentalism, need for closure, errors, brain activity, N400 component

## Abstract

Examining the relationship between brain activity and religious fundamentalism, this study explores whether fundamentalist religious beliefs increase responses to error-related words among participants intolerant to uncertainty (i.e., high in the need for closure) in comparison to those who have a high degree of toleration for uncertainty (i.e., those who are low in the need for closure). We examine a negative-going event-related brain potentials occurring 400 ms after stimulus onset (the N400) due to its well-understood association with the reactions to emotional conflict. Religious fundamentalism and tolerance of uncertainty were measured on self-report measures, and electroencephalographic neural reactivity was recorded as participants were performing an emotional Stroop task. In this task, participants read neutral words and words related to uncertainty, errors, and pondering, while being asked to name the color of the ink with which the word is written. The results confirm that among people who are intolerant of uncertainty (i.e., those high in the need for closure), religious fundamentalism is associated with an increased N400 on error-related words compared with people who tolerate uncertainty well (i.e., those low in the need for closure).

## Introduction

Religious fundamentalism has significantly shaped world history and continues to influence individual attitudes and behaviors (e.g., Emerson and Hartman, 2006; Paloutzian and Park, 2014). It represents a distinctive attitude of certainty as to the ultimate truth of one’s religious beliefs (Altemeyer and Hunsberger, 1992, 2004). Most researchers emphasize its contributions to conflicts around the world (Moaddel and Karabenick, 2008, 2013; Ginges et al., 2009; Neuberg et al., 2014). Recently, however, researchers have demonstrated that it provides individuals with a sense of meaning in life and that it offers relief from distress and uncertainty (Hood et al., 2005; Williamson and Hood, 2014; Phillips and Ano, 2015; Kossowska et al., 2016). It also fosters self-regulation and, more specifically, self-control (e.g., McCullough and Willoughby, 2009; Rounding et al., 2012; McCullough and Carter, 2013). Self-control is needed to bring one’s behavior in line with fundamentalist rules and standards. One possible way to increase the alignment between rules and behavior is to monitor the environment as well as one’s own behaviors for errors (Baumeister and Heatherton, 1996). Although previous studies focused on monitoring one’s own errors (Inzlicht et al., 2009; Senderecka et al., unpublished), in this paper we examined participants’ responses to error-related words. We tested the hypothesis that religious fundamentalism is related to increased sensitivity to error-related words, especially among people who are intolerant of uncertainty, i.e., those high in the need for closure (NFC, Kruglanski, 1989). We test this claim at the electrophysiological level. We claim that religious fundamentalism causes people who are intolerant of uncertainty to care more about errors. In that way, our study contributes to the existing literature on the relationship between religiosity and self-control.

### Religious Fundamentalism, Self-Control, and Error Sensitivity

Religious fundamentalism is here taken to be a collection of infallible beliefs or principles that provide guidance regarding how to obtain salvation. Religious fundamentalists believe in the superiority of their religious teachings, and in a strict division between righteous people and evildoers (Altemeyer and Hunsberger, 1992, 2004). This belief system regulates religious thoughts, but also all conceptions regarding the self, others, and the world. It is a “meta-belief” – a worldview that provides an absolute foundation for determining what to do in various particular situations and for how to live in general (Altemeyer and Hunsberger, 1992; Koltko-Rivera, 2006). Therefore, it helps provide a sense of coherence and control, and it helps to reduce ambiguity about the world (Altemeyer and Hunsberger, 1992; Pargament, 2002; Hood et al., 2005; Brandt and Reyna, 2010; Williamson and Hood, 2014; Phillips and Ano, 2015; Kossowska et al., 2016).

Indeed, research suggests that fundamentalists tend to believe that they themselves control the outcomes of various events, while non-fundamentalists are more sympathetic to the view that such outcomes are influenced by others or by chance (Silvestri, 1979; Tipton et al., 1980; Furnham, 1982). Moreover, in their meta-analysis, McCullough and Willoughby (2009) demonstrated that religious fundamentalism not only increases the subjective feeling of control, but that it also fosters self-regulation and, more specifically, self-control. Self-control is the ability to direct one’s behavior in a way that is in line with rules and standards that are meaningful (Baumeister and Heatherton, 1996). By means of self-control, one may refocus attention, alter one’s mood or emotional state, overcome fatigue, resist temptation, or in various other ways change one’s state of mind or one’s actions (Geyer and Baumeister, 2005). Baumeister and Exline (1999, 2000) have argued that in order to behave virtuously, such as avoiding vice or sin, self-control is necessary. Indeed, research has shown that religious fundamentalism is positively related to temptation resistance, the endurance of discomfort, the ability to delay gratification, and response accuracy (e.g., Inzlicht and Tullett, 2010; Laurin et al., 2012; Rounding et al., 2012; for a review see McCullough and Willoughby, 2009).

One of the important components of successful self-control is monitoring one’s performance, particularly for errors (Baumeister and Heatherton, 1996). This is due to the fact that in order to achieve important goals, people need be aware of what they are doing and of how that behavior compares to the standard for which they are striving. In addition, errors are aversive. The commission of errors might also be threatening, as they place the individual in unknown danger (Hajcak and Foti, 2008; Hajcak, 2012; Spunt et al., 2012). Errors provide both the suggestion and motivation for correcting one’s behavior, and their correction can help to guide behavior toward a desired goal state (Baumeister et al., 1994; Baumeister and Heatherton, 1996). Thus, especially under conditions of uncertainty, highly religious fundamentalist people should efficiently monitor their behavior for errors. Only by being aware of making mistakes can they direct their behavior according to the standards expressed in fundamentalist worldviews. Therefore, they should increase sensitivity to error-related content.

However, the empirical support for this claim is limited and mixed. For example, using neural correlates of performance monitoring, i.e., an event-related potential (ERP) called the error-related negativity (ERN; Falkenstein et al., 1990; Gehring et al., 1993), Inzlicht et al. (2009), Inzlicht and Tullett (2010), Good et al. (2014) reported that religion causes people to care less about making errors in the Stroop task. However, Senderecka et al. (unpublished), using a stop signal task, found significantly larger error-related negativity, correct-related negativity, and post-error positivity components in high (vs. low) religious fundamentalists, pointing to their increased engagement in error response monitoring. In explaining these inconsistencies, the researchers argue that the crucial component of religious beliefs that may facilitate self-control, thus increasing error responses, is the belief in a punishing but not forgiving God (see also Shariff and Norenzayan, 2011; Shariff and Rhemtulla, 2012). If prominent in religious fundamentalists’ minds, this belief may make them more aware of the discrepancies between their imperfect performance and rigorous standards of correct behavior. In studies by Senderecka et al. (unpublished), religious fundamentalism was measured by the Pro-Fundamentalism Scale (Altemeyer and Hunsberger, 1992), since it includes items directly related to the concept of God’s punishment and the necessity to obey His strict laws. On the other hand, studies by Inzlicht et al. (2009) focused on measuring or priming religious, but not fundamentalist, beliefs.

In the studies mentioned above, the researchers have focused on monitoring one’s own activity and one’s own responses to making errors. However, we suggest that the general sensitivity to error-related events or words may also be considered an important mechanism through which fundamentalism facilitates self-control. Errors are not only aversive, but they may provide important signals that external events pose a threat to the fundamentalists’ worldview. Therefore, religious fundamentalists, especially those for whom certainty is especially important, should be more sensitive to error-related events as potential signals of threat. We claim that increased sensitivity to error-related words poses the threat to the sense of certainty.

### Overview of the Study

We tested this hypothesis in an experimental study in which we measured individual differences in religious fundamentalism. As the beneficial function of religious fundamentalism has been mainly demonstrated under conditions of stress or uncertainty (Hood et al., 2005; Williamson and Hood, 2014; Phillips and Ano, 2015; Kossowska et al., 2016), or among those who are especially sensitive to distress and uncertainty (Brandt and Reyna, 2010; for a review see, Pargament, 2007), we focused on individual differences in intolerance of uncertainty, called the need for closure (NFC, Kruglanski, 1989)^1^. NFC constitutes a fundamental epistemic motive behind how people process information from the social environment. It refers to the level of an individual’s desire for clear and certain explanations, and importantly, those explanations are desired over and above the person’s willingness to accept ambiguity and uncertainty (Webster and Kruglanski, 1994). Thus, intolerance of uncertainty is a fundamentally important element of cognitive closure. Recently, this claim has been supported by neuropsychological analyses (Kossowska et al., 2014). We predicted that high religious fundamentalism would be related to increased sensitivity to error-related events among those who are intolerant of uncertainty (i.e., high in NFC), but not among those who tolerate uncertainty (i.e., low in NFC).

We hypothesized that the expected enhanced response to errors may be observed neurophysiologically. Research using ERP has shown that the N400, a negative-going deflection peaking around 400 ms poststimulus onset, is primarily involved in processing information related to semantics and violations of meaning (Kutas and Hillyard, 1984; Kutas and Federmeier, 2000). Although initially discovered when participants examined incongruent words in sentences, researchers have recently demonstrated that N400 is also elicited by incongruent words and knowledge about the self and the world (van den Brink et al., 2001). A social psychological interpretation of this finding is that the N400 encodes the violation of expectancy which arises from people’s assumptions about the world and the self (van Berkum et al., 2009; Huang et al., 2014). Due to its important role in the processing of assumption violations and expectancy, the N400 is a component of ERP that may also encode information related to emotional or social conflict (White et al., 2009; Chen and Spence, 2010). The stronger the conflict, the higher the N400 (more negative mean/peak voltages).

We expected that religious fundamentalism among people intolerant to uncertainty would be related to more intense processing of error-related stimuli (increased N400 amplitude) compared to those who tolerate uncertainty well. As explained above, errors violate the religious fundamentalists’ expectations of being correct in their behaviors and always following the rules. Therefore, error-related events are signals that there is a possible threat that the rules may be broken, which can lead to misbehaviors, or at least to deviations from valued standards. Thus, religious fundamentalists should be sensitive not only to their own errors, but also to any event in the environment that suggests possible errors. To test this assumption, while participants were performing an emotional Stroop task (EST), we recorded the electroencephalographic activity of the brain (EEG) (McKenna and Sharma, 1995). In the EST, participants read the words, while being asked to name the color of the ink with which the words were written (McKenna and Sharma, 1995). Klein (1964) and Dalgleish (2005) have argued that the EST produces interference in information-processing not only due to the semantic meaning associated with the word, but also due to the emotional content reflecting an individual’s implicit attitudes, motivations, and emotions. Throughout numerous variations of the EST, it has been consistently found that people have difficulty ignoring the meaning of a word while naming the color in which it is written (Johnson and Hasher, 1987; Salo et al., 2001). It is then not surprising that when the words relate directly to the participants, higher response latencies result (Logan and Goetsch, 1993). In our experiments we use neutral words, as well as words related to uncertainty, errors, and pondering. We expected an interference effect for error-related words, but not for words related to uncertainty or pondering. As only words related to errors are inconsistent with fundamentalist views of correct behavior, they may be particularly unexpected, emotionally salient, or otherwise attention grabbing, and thus they might call for intensified processing of meaning, thereby elevating the N400.

The experimental procedure followed the ethical principles described in the 1964 Declaration of Helsinki (World Medical Organization, 1996) and was approved by the Research Ethic Committee at the Institute of Psychology, Jagiellonian University. The written informed consent was obtained from the participants of this study. The study was run in 2013.

## Materials and Methods

### Participants

Before the experiment, a group of 295 students (242 women, 53 men, *mean age* = 24.75, *SD* = 4.58) filled out the short, Polish version of the NFC Scale (Webster and Kruglanski, 1994; Kossowska et al., 2012). The NFC scores (α = 0.72; *M* = 3.45; *SD* = 0.65), roughly normally distributed, were used to create two groups with higher (>90th percentile) and lower (<10th percentile) psychometric NFC scores. Thus, only 67 participants were invited to the experiment (41 women, *mean age* = 24.36, *SD* = 5.48) and only 42 accepted our invitation and show up in the laboratory (34 women, *mean age* = 23.60, *SD* = 4.78). For purpose of this study we decided to preselect participants based on their NFC levels as the effect we study is expected to occur only under uncertainty or among people sensitive for uncertainty. In addition, due to the specificity of EEG studies (they are costly, time-consuming and difficult for participants), we intentionally used small sample sizes (see Preacher et al., 2005).

All of the participants had normal or corrected to normal vision and normal hearing. All of the participants reported that they did not have any neurological or psychiatric disorders, including drug abuse, and that they were not on any medications during the experiment. They signed an informed consent and received 20 PLN (roughly €5) for their participation. Data from three participants were not included in the analysis because on pre-processing data stage problems with recording and due to excessive muscle artifacts reviled in them. The remaining 39 participants^2^ (33 women, 6 men) had a mean age of 23.67 (*SD* = 4.94). All of them self-reported as being religious, and as having been brought up in Christianity.

### Measures/Procedure

The experiment was run in a sound-attenuated cabin. At the start of the experiment, participants filled out the Religious Fundamentalism Scale (RFS; Altemeyer and Hunsberger, 1992) which assesses one’s attitude toward religious belief in a way that is independent from any specific religion and any particular set of religious beliefs. This scale defines religious fundamentalism along four dimensions: (1) the belief that there is a single set of religious teachings containing the fundamental, basic, intrinsic, inerrant truth about the deity and humanity; (2) this essential truth stands in opposition to evil, which must be actively fought; (3) the truth is to be followed in our current day according to the fundamental practices of the past; and (4) people who succeed in following these fundamental teachings have a special relationship with the deity. The scale contains 12 statements that participants assess on a 5-point Likert scale from 1 = “Strongly Agree” to 5 = “Strongly Disagree.” Higher scores indicate a higher level of religious fundamentalism. The reliability of the scale obtained on the current sample was acceptable (Cronbach’s α = 0.70, *M* = 2.89, *SD* = 0.87). Participants then once again filled out the NFC Scale to check test–retest stability [*r*(39) = 0.91, *p* < 0.001]. All participants stayed within previous category of high and low NFC.

Participants were then asked to perform the EST, as adapted from Smith and Waterman (2003). This computerized version of the EST consisted of a series of words (neutral terms and those related to errors, pondering, and uncertainty) printed in one of four different colors (yellow, red, green, or blue), and grouped in four blocks. The words were selected based on a pilot study. The words used in the procedure are presented in **Table 1**. Participants were instructed to name the font color of a presented word and to neglect its meaning. After 28 practice trials (for the training session numerals – one, two, etc. – were used instead of the words), participants were given 40 experimental trials per block. In each trial, a word was presented for 200 ms; the maximum time for a response was restricted to 2200 ms. The blocks were presented in a random order. A 22″ computer screen was placed approximately 70 cm away from the participants. The procedure was programmed in PsychoPy software. Both reaction times (RT) and identification accuracy related to the emotional words (error, uncertainty, and pondering) are compared to the scores related to neutral words, and the difference (interference score) is understood to be the result of interference arising from the words’ emotional content (Wentura et al., 2000; Larsen et al., 2006).

**Table 1:** Words used in the emotional Stroop task (EST).

Error trails	Uncertainty trails	Pondering trails	Neutral trails
Blunder	Anxiety	Options	Piece
Error	Uncertainty	Problem	Title
Punishment	Apprehension	Examination	Window
Mistake	Doubt	Reflection	Gate
Defeat	Risk	Thinking	Box
Slip	Ambiguity	Discussion	Hour
Rebuke	Unknown	Hesitation	Curtain
Critique	Instability	Contemplation	Teaspoon
Reprimand	Variability	Philosophy	Umbrella

### Electroencephalography Recording

The EEG signal was recorded during the participants’ completion of the EST using a Biosemi Active Two device equipped with 64 active electrodes placed on a 10–10 headcap, and two electrodes placed on the left and right mastoids for off-line linked mastoid reference. Additional four leads were located above and below the right eye and in the external canthi of both eyes. The signal was sampled at 256 Hz frequency and filtered using 46 Hz low-pass and 0.1 Hz high-pass zero-phase digital filters. Oculomotor correction was carried out using Least Mean Squares (LMS) regression (Gómez-Herrero, 2007). After data epoching (-100 to 800 ms) a 100 μV rejection threshold was set to exclude segments contaminated with potential artifacts.

### Event-Related Potentials

Evoked potentials were analyzed time-locked to the stimuli onset with baseline correction using the -100 to 0 ms epoch. The N400 component was defined as averaged activity from the POz, CPz, and Pz sites in the 300–500 ms time window. Differences of the resulting N400 values between uncertainty, errors, and ponder blocks and the neutral block were considered. As N400 is a negative component, the lower the index means the stronger interference.

## Results

The emotional Stroop interference effect for ERP, RT and accuracy was computed as the difference between the experimental trials and the neutral words. For each experimental measure (separately for all trial types, i.e., pondering, uncertainty and error) the respective value related to neutral words were subtracted. The obtained results were used as dependent variables in all further analysis. To test our hypothesis, we ran a moderation model using PROCESS program (Hayes, 2013, model 1, bootstrap 10,000) with religious fundamentalism as a predictor and NFC as a moderator, for each of the dependent variables. The moderator was coded (1 high NFC/0 low NFC). Religious fundamentalism was centered.

### Behavioral Results

We did not find any main effects of religious fundamentalism or NFC, nor did we find an interaction between them for interference index related to uncertainty, pondering and error at the behavioral level of analysis, neither for RTs (*p*s > 0.3) nor accuracy (*p*s > 0.8).

### Electrophysiological Results

**FIGURE 1:**
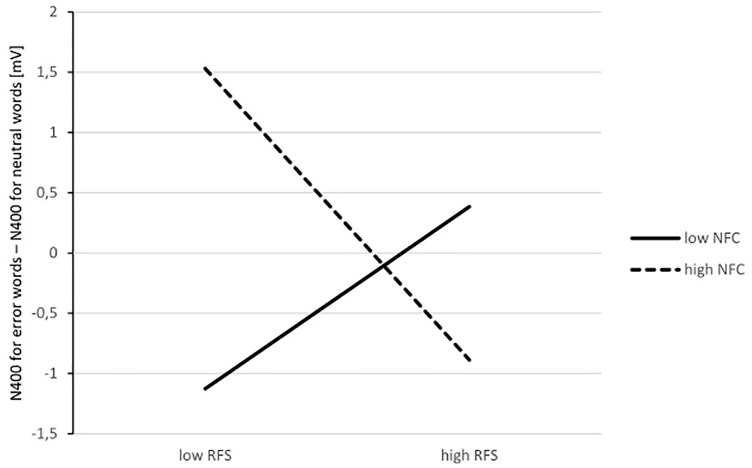
Interaction between need for closure (NFC) and Fundamentalism on Stroop effect index (N400 for error words – N400 for neutral words). The more negative score, the stronger interference. For religious fundamentalism –1 SD, +1 SD values were used.

**FIGURE 2:**
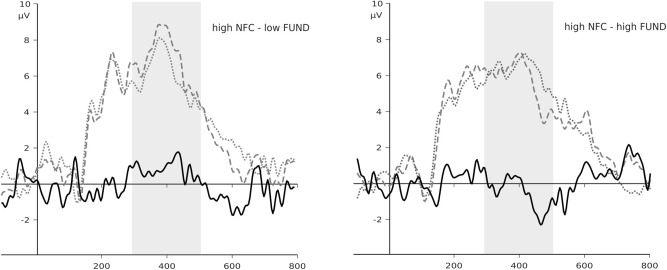
Event-related potential (ERP) plots for high NFC group and median-split of fundamentalism scores averaged from the POz, Pz, CPz electrodes. The N400 component is defined as a difference (black solid line) between average activity within the 300–500 ms window for error-related (dashed lines) minus neutral (dotted lines) words. Gray polygons marks the time window of interests (300–500 ms), relative to words onset. Despite the absolute differences between values, the statistical significance was found only for the high NFC slope and not for the low NFC. Without these statistical parameters, the ERP plots may be misleading on a first sight. Moreover, the presented plots are provided only for illustrative purposes, and were created with median split of the variables. The results that are considered in the manuscript were computed using continuous variables.

We did not observe any significant main effects of religious fundamentalism or NFC nor did we find interactions between them in interference effects for words related to uncertainty and pondering (*R*^2^ = 0.01, *b* = -0.62, *p* = 0.620, 95% CI [-3.12, 1.88]; *R*^2^ = 0.01, *b* = -0.63, *p* = 0.603, 95% CI [-3.05, 1.80], respectively). However, while we found no significant main effect of religious fundamentalism in interference effect for words related to error (*b* = 0.87, *p* = 0.254, 95% CI [-0.65, 2.38]), there was a significant effect of NFC (*b* = 7.19, *p* = 0.028, 95% CI [0.82, 13.56]) on N400 interference effect for words related to error. Furthermore, the results revealed a significant interaction between religious fundamentalism and NFC (*R*^2^ = 0.12, *b* = -2.25, *p* = 0.038, 95% CI [-4.37, -0.14]) on N400 interference effect for words related to error. We then performed simple slope analyses because we were interested in the relationship between religious fundamentalists and N400 interference effect for words related to error for low and high NFC separately. The analyses indicated that religious fundamentalism was marginal negatively related to N400 interference effect for words related to error for high NFC individuals (*b* = -1.39, *p* = 0.064, 95% CI [-2.86, 0.09]), and was positively but non-significantly related for low NFC participants (*b* = 0.87, *p* = 0.254, 95% CI [-0.65, 2.38]). We present the illustration of the interaction between NFC and Fundamentalism on Stroop effect index based on real calculations, with provided by Process Macro data for plots on **Figure 1**. In addition, for illustrative purpose we present ERPs’ plots for high NFC group on **Figure 2**.

## Discussion

Researchers claim that there is a link between self-control and religion (McCullough and Carter, 2013). They suggest that religion helps promote delayed gratification, prosocial tendencies, and other socially adaptive behaviors (Baumeister and Exline, 2000). Explaining the link between self-control and religion, they suggest that by making salient the belief in an ever-watchful God (Norenzayan and Shariff, 2008) religion may in effect encourage self-monitoring (see McCullough and Willoughby, 2009). This belief may also increase concerns with punishment, by focusing one’s attention on the image of a punishing and not forgiving or loving God (Shariff and Norenzayan’s, 2011). Religious concepts may also bring reputational concerns to the fore and this increased salience may in turn promote more self-monitoring (Rounding et al., 2012). The empirical support for these claims is, however, very limited and mixed.

In this preliminary study we argue that religious beliefs, in a fundamentalist form, are linked to monitoring for errors, an important component of self-control (Baumeister and Heatherton, 1996). Errors are aversive as they distort the pleasant and stable pictures one might have of the self and the world, and they thus destroy predictability. In addition, for people with highly fundamentalist views that include standards for correct behaviors and suggest strict rules to follow and severe punishments for deviation from them, errors should pose a special threat. A fundamentalist view may also make people more aware of the discrepancies between their imperfect performance and the rigorous standards of correct behavior. We claim that not only errors related to one’s own performance, but also those signaling possible dangers in environment, should play the same role. To further develop this idea, we focused on the link between religious fundamentalism and responses to error-related words.

Thus, the aim of the current study was to examine whether religious fundamentalism is related to altered self-control evinced as sensitivity to error-related words among participants highly intolerant of uncertainty (high NFC) compared to those with a high tolerance of uncertainty (low NFC). The results of our study indicate that religious fundamentalism and intolerance to uncertainty differentiate electrophysiological measures related to error monitoring. In particular, we found significantly larger error-related brain activity in the N400 for religious fundamentalism in the high NFC group (decreased interference index), although N400 decreased for religious fundamentalism among participants low in NFC. Behaviorally, however, there were no significant differences between these groups on RTs or accuracy. Thus, our results may reflect an increased orienting response toward error-related stimuli, related to autonomic nervous system activity (e.g., Hajcak et al., 2003; Wessel et al., 2011).

Many studies have demonstrated the palliative function of religious beliefs, also in fundamentalist forms, under conditions of uncertainty (e.g., Inzlicht et al., 2009; Inzlicht and Tullett, 2010). Indeed, across samples as diverse as members of community churches, college students, and the hospitalized elderly, researchers have found that strong religious belief is connected with beneficial outcomes related to stress, such as better overall mental health and higher reported levels of stress-related growth (for a review see Pargament, 2007). To build on this previous research, in our study we measured intolerance to uncertainty understood as the need for closure, believing that among people highly intolerant to uncertainty, religious fundamentalism may play this defensive role. In this regard, increased sensitivity to error-related events may be considered a defensive mechanism. Detecting errors may allow one to bring their behavior in line with fundamentalist rules and standards.

Our findings are in line with a recent study by Senderecka et al. (unpublished) showing that high levels of religious fundamentalism were associated with a larger ERN/CRN amplitude, indicating larger monitoring for error making. Thus, these researchers suggest that people who are highly religiously fundamentalist were more aware of their errors or found their errors more motivationally salient and attention-engaging. It is worth stressing that this study shows that when people largely focus on God’s punishment and the necessity to obey His strict laws (measured by the Pro-Fundamentalist Belief Scale), discrepancies between expected and actual outcomes of their behaviors evoke highly aversive cognitive conflict. In such situations, both self-monitoring for errors and sensitivity to events that may potentially signal errors in the environment become especially important.

In our study, we used the Religious Fundamentalism Scale (Altemeyer and Hunsberger, 1992), and not just some items directly related to the concept of God’s punishment and the necessity to obey His strict laws. The studies were, however, conducted in Poland where Catholicism places considerable value on rule-following (Strzelczyk, 2016). Thus, it is highly likely that our religious participants had this image of God in mind.

An alternative interpretation could be also mentioned, which relates to the association of the N400 component to the semantic integration within a more general context. People scoring high on fundamentalism and NFC could be characterized with a general tendency to avoid information that relates to uncertainty, conflict or errors. Such defense tendency would make any error-related semantic content less available in their cognitive system. Observed increase of N400 response to presentation of such words could be a manifest of incongruity with their self-concept, where there is a limited acceptance of conflicting tendencies and committing errors. The attempts to filter out error-related information would also result in higher emotional incongruity after being directly exposed to such evaded words. We did not, however, observed significant effects on N400 by uncertainty-related words, which makes above interpretation more speculative.

There are, however, a few important limitations of the study. The study shows a correlation between religious fundamentalism and response-related brain activity; however, the causal direction of this relationship is unclear. Further research is needed to determine whether a fundamentalist mindset causes overactive performance monitoring or, on the contrary, excessive behavioral monitoring leads to religious fundamentalism. In addition, fundamentalism was studied on quite a homogeneous sample of young Polish Catholics. Thus, studying this effect across religions and cultures will likely yield valuable insights.

Next, although small, low-powered studies are endemic in neuroscience, they are also problematic (Larson and Carbine, 2017). It was recently recognized that low sample size of studies, small effects or both, lead to low statistical power that negatively affects the probability that a nominally statistically significant finding actually reflects a true effect. For example, Button et al. (2013) demonstrated a small sample size is responsible for a low probability of replication, exaggerated estimates of effects when a statistically significant finding is reported, and poor positive predictive power of small sample effects. In addition, due to small sample size we could not reliably test the three-way interaction between the word type, NFC and religious fundamentalism that would allow us to verify the hypothesis that high religious fundamentalism accompanying high intolerance of uncertainty is related to stronger reactions to error-related words in comparison to ponder- or uncertainty-related words. The results of such underpowered testing would be simply inconclusive. It is worth noticing, however, that we did not formulate the hypothesis in that way. We expected an interference effect for error-related words, but not for words related to uncertainty or pondering. Thus, we focused on NFC × religious fundamentalism interaction tested for interference in words related to error, pondering, and uncertainty separately. This is of course not optimal strategy, but reasonable having small sample available. Therefore, the results should be treated with some caution and replications of the results would be of great value. At the same time, we are sure that the study was carefully prepared according to the standards in EEG research, i.e., the time ranges and electrodes were selected based on typical values in the N400 literature, and were not data- or significance-driven. Thus, it may be treated as a good starting point for future analysis.

Finally, in order to fully understand the psychological processes underlying fundamentalist beliefs, it is important to understand both their detrimental and their beneficial cognitive functions. It is important to keep in mind that such research is related to neither assertions of, nor denials of, the truth value of such beliefs. The current study is important in that it contributes to our understanding of the link between religious fundamentalism and self-control.

## Author Contributions

MK developed the rationale of the study, the study concept, and wrote the manuscript. PS and GC contributed in data collection. PS and MW contributed in data analyses and its interpretation. All authors contributed in study design, critically read the manuscript and provided comments that helped improve its final version, and approved the final version of the manuscript for submission.

## Conflict of Interest Statement

The authors declare that the research was conducted in the absence of any commercial or financial relationships that could be construed as a potential conflict of interest.

## Footnotes

1 There are many constructs related to uncertainty as intolerance of uncertainty (Buhr and Dugas, 2002), tolerance of ambiguity (Budner, 1962) or uncertainty orientation (Sorrentino et al., 1992). They differ each other as it was demonstrated in many studies. We deliberately chose the NFC as it constitutes a fundamental epistemic motive underlying how people approach and process social information. From social psychology perspective NFC is important construct with solid theoretical background and well established social and cognitive effects systematically tested for over 30 years (see Roets et al., 2015). In addition, the NFC scale is well tested in cultural context of the study. It will be important, however, to test all of these constructs in religious context but it should be the goal of future studies.

2 following the suggestion of study being underpowered we have performed the statistical power *post hoc* analysis (using G^∗^Power 3.1.9.2, Faul et al., 2009) to verify how likely it was to observe a statistically significant interaction on the N4 in our study. To this aim, we focused on paradigm in which the N4 component is studied aside of directly linguistic context. The analysis was based on the sample size we used in our experiment (*N* = 39) and alpha was defined as classical 0.05 level. The effect size was based on the literature when reported N400 effect is either medium or large. To be secure we used in our *post hoc* analysis medium one, so Cohen’s *f* = 0.39. Given our sample size it is revealed we had 97% power to detect a true N4 component.
